# Analysis of the association between non-alcoholic fatty liver disease and mortality in United States adults

**DOI:** 10.3389/fnut.2024.1502671

**Published:** 2024-11-22

**Authors:** Fen Rong, Yiyi Mai, Lujia Shou, Xinya Zhu, Meiyue Li, Liuchen Zhu, Xiuli Sun, Xinhua Zhao

**Affiliations:** ^1^School of Public Health, Shanghai University of Traditional Chinese Medicine, Shanghai, China; ^2^School of Traditional Chinese Medicine, Shanghai University of Traditional Chinese Medicine, Shanghai, China; ^3^Library of Shanghai University of Traditional Chinese Medicine, Shanghai, China

**Keywords:** non-alcoholic fatty liver disease, all-cause mortality, CVD mortality, fatty liver index, serum uric acid

## Abstract

**Background:**

Non-alcoholic Fatty Liver Disease (NAFLD) is a prevalent condition characterized by the accumulation of fat in the liver, often linked with increased risk for multi-systemic diseases. This study aims to investigate the relationship between NAFLD and mortality, particularly all-cause and cardiovascular mortality, among United States adults.

**Methods:**

Data from the National Health and Nutrition Examination Survey (NHANES) were utilized, encompassing 80,312 participants from 2003–2004 to 2017–2018. After exclusions for incomplete data, potential other liver diseases, and significant alcohol consumption, the final analytical cohort included 32,698 participants. The Fatty Liver Index (FLI), a non-invasive diagnostic tool, was used to identify NAFLD. Covariates included demographic characteristics, lifestyle factors, and biochemical parameters. Survival analysis was conducted using a weighted Cox proportional hazards regression model to quantify the impact of NAFLD on mortality.

**Results:**

The study revealed that NAFLD was significantly associated with increased risks of all-cause and cardiovascular disease (CVD) mortality. The hazard ratios (HRs) from the survival analysis consistently indicated a higher risk among participants with NAFLD compared to those without. Subgroup analyses further confirmed the association, with notable exceptions in certain subgroups such as those with high school education and diabetes. Additionally, a nonlinear relationship between serum uric acid (SUA) levels and mortality risk was identified among NAFLD participants.

**Conclusion:**

Non-alcoholic Fatty Liver Disease is a significant risk factor for all-cause and CVD mortality in US adults. The findings underscore the importance of early detection and intervention for NAFLD to mitigate its impact on public health. Further research is needed to explore the complex interactions between NAFLD, SUA levels, and mortality, particularly in high-risk subgroups.

## 1 Introduction

Non-alcoholic fatty liver disease (NAFLD) which is typically diagnosed by liver biopsy is defined as a degree of steatosis of the liver in the absence of excessive alcohol consumption and other known causes (1, 2) and has a global prevalence increasing from 25.5% in or before 2005 to 37.8 in 2016. The prevalence is higher in the United States, reaching 47.8%. The harm of NAFLD is not only limited to liver-related morbidity and mortality, but also a link to the increased risk of multisystem disease (3–5). If not managed properly, NAFLD could be a progressive disease that leads to serious liver fibrosis, cirrhosis, even liver cancer. There is a strong association demonstrated between NAFLD and the elevated risk of developing type 2 diabetes, obesity, sex, cardiovascular diseases and chronic kidney disease (6–8). Therefore, NAFLD has emerged as a significant global public health problem bringing us heavy clinical and economic burden calling for our great concern (9, 10).

Plenty of studies showed NAFLD was closely associated with the increased all-cause mortality, fatal or non-fatal cardiovascular diseases (CVDs) events (11, 12). In the United States, the age-standardized mortality in the participants with NAFLD has been increasing at an annual rate of 7.8% over the past decade and it is projected to reach 1.83 million deaths related to NAFLD annually by 2030 (13). Among the population with NAFLD, CVDs is identified as the second leading specific cause of death after cirrhosis (14). NAFLD is also associated with a broad range of cardiovascular symptoms including subclinical atherosclerosis, atrial fibrillation, cardiac conduction defects, aortic-valve sclerosis and left ventricular diastolic dysfunction, etc. (15, 16). However, the heterogeneity of NAFLD risks across various demographic factors, dietary habits, and metabolic status and whether NAFLD is an independent risk factor for CVDs is still under debate (17–19). This study aims to delve into the relationship between NAFLD and all-cause and CVD mortality in United States adults.

Yusha (20) and Jie pan (21) conducted analyses on the association between NAFLD and mortality, but notably omitted some key risk factors, e.g., daily eating habit and physical activity. Furthermore, they did not specifically focus on the subpopulation diagnosed with NAFLD complicated by elevated serum uric acid (SUA) levels, which have been demonstrated closely associated with increased risk of all-cause and CVDs mortality in overall population (22, 23). A nonlinear dose–response curve was reported to reveal this association (24, 25). In this study, we also conducted a similar analysis to investigate the association between SUA and mortality in NAFLD participants.

## 2 Methods

### 2.1 Data source

The analysis used the database of National Health and Nutrition Examination Survey (NHANES) which is a program of studies conducted by the Centers for Disease Control and Prevention (CDC). It is designed to assess the health and nutritional status of adults and children in the United States. The database consists of data collected from interviews and physical examinations and is open to the public (26).

In our analysis, we focused on two key mortality outcomes: all-cause mortality defined as the deaths from any cause, and CVD mortality specifically referring to the deaths caused by heart disease and cerebrovascular disease (27).

### 2.2 Study participants

**Figure 1:**
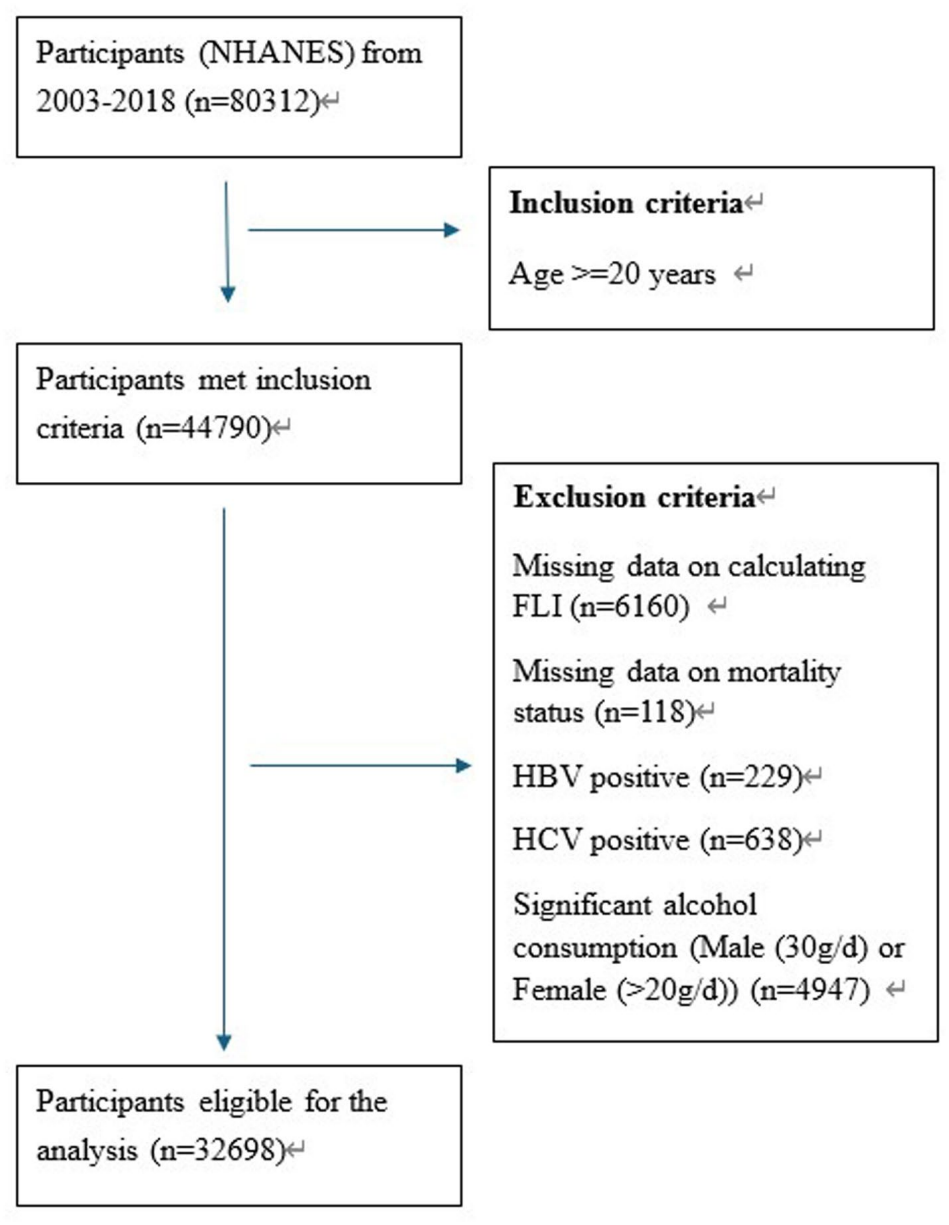
Eligible participant screening process.

We included 80,312 participants in total from 8 cycles of survey (2003–2004 to 2017–2018) of NHANES. According to the adult age criteria utilized in other studies (28), we only included the participants of 20 years old and above (*n* = 44,790). In consideration of the data integrity, 6,160 participants were excluded due to missing data on the calculation of Fatty Liver Index (FLI) and 118 participants were excluded due to missing data on mortality status. To further eliminate the liver diseases potentially caused by viral hepatitis and alcohol consumption, 229 with HBV positive, 638 with HCV positive and 4,947 participants with significant alcohol consumption were also excluded. Finally, there were 32,698 participants eligible for our analysis. More details about the participants screening process are shown in Figure 1.

### 2.3 Diagnosis of NAFLD

Although as the gold standard of NAFLD diagnosis (29), liver biopsy is not widely used in the first-line screening due to its invasive nature (30). Numerous non-invasive methods, e.g., serum marker and ultrasound are introduced to screen NAFLD. As an algorithm, fatty liver index has been in use in practice for over 17 years and demonstrated the accuracy comparable with other diagnostic methods (31, 32).

Fatty liver index was calculated as following formula:


$$\begin{array}{l} FLI=\\ \left(\begin{array}{l} e^{0.953\times Ln\left(TG\right)}+0.139\times BMI+0.718\times \\ Ln\left(GGT\right)+0.053\times WC-15.745 \end{array}\right)÷\\ \left(\begin{array}{l} 1+e^{0.953\times Ln\left(TG\right)}+0.139\times BMI+0.718\times \\ Ln\left(GGT\right)+0.053\times WC-15.745 \end{array}\right)\times 100 \end{array}$$

Where TG (mg/dL), GGT (U/L), BMI (kg/m^2^), and WC (cm) stand for triglycerides, gamma-glutamyl transferase, body mass index and waist circumference, respectively.

The FLI values (33, 34) ranged from 0 to 100 and were categorized into three levels: <30 no steatosis, 30–59 in intermediate state and ≥ 60 with hepatic steatosis (34, 35). Therefore, in our analysis, it was diagnosed as NAFLD when FLI ≥ 30 if other known causes of liver diseases could be ruled out (36).

### 2.4 Data source of mortality status

The information of follow-up and mortality could be obtained from the Public-Use Linked Mortality Liles where the data through December 31, 2019 linked to the participants in NHANES. By referring to the codes of the International Statistical Classification of Diseases and Related Health Problems, we were able to figure out the cause of death.

### 2.5 Selected covariates

Numerous studies have underscored the relationship between NAFLD and mortality, with this association often being influenced by some demographic characteristics. Therefore, to further explore the potential association, some covariates were identified and included in our analysis.

The covariates included age (years: ≤ 39, 40–59, ≥60), sex, race and ethnicity (Mexican American, non-Hispanic Black, non-Hispanic White, and other including other Hispanic, other non-Hispanic, and non-Hispanic multiple races), education level (<High school, High school and Some college or above), smoking status, daily eating frequency (DEF) which was measured by the frequency of eating occasion during day 1 and day 2 (37), Body mass index (BMI: <30.0, ≥30.0), diabetes, hypertension, physical activity measured by Metabolic equivalent (MET) intensity score (VLPA: <150 MET-min/week, LPA: 150–960 MET-min/week, MPA: 961–1800 MET-min/week and HPA: >1800 MET-min/week), alanine aminotransferase (ALT in U/L), aspartate aminotransferase (AST in U/L), high-density lipoprotein (HDL in mmol/L) and low-density lipoprotein (LDL in mmol/L).

### 2.6 Statistical analysis

In this study, we aimed to explore the long-term increased risk associated with NAFLD by performing a survival analysis on NHANES data during 2003–2018. The effect size was measured using hazard ratio (HR) by comparing those with NAFLD to those without.

According to the official guidance by NHANES, survey weights were utilized in NHANES to account for the complex survey design, including oversampling, survey non-response and post-stratification adjustment (38). To combine two or more 2-year cycles of the continuous NHANES data, the appropriate weights should be constructed to ensure the estimates accurately represented each survey cycle.

For demographic characteristics, the summary for categorical variables was presented by frequency with percentage and medians with interquartile range (IQR) for continuous variables given their non-normal distribution. The comparison between NAFLD and Non-NAFLD was performed by using rank sum test, e.g., Kruskal-Wallis rank test for continuous variables and Chi-square test for categorical variables, with corresponding *p* values reported. In the survival analysis, we applied weighted Cox proportional hazards regression model to examine the link between NAFLD and all-cause mortality and CVDs mortality. The results were presented as HR with 95% Cis and Kaplan–Meier survival curves. Additionally, forest plots were also presented to detect the heterogeneity of this association across various demographic characteristics.

To detect the dose–response relationship between SUA and all-cause and CVDs mortality, a restricted cubic spline with four knots was employed. To ensure the robustness of our outcomes, the sensitivity analyses were also conducted: (1) only considered the participants with hepatic steatosis (FLI ≥ 60) as NAFLD to perform survival analysis; (2) excluded the participants who died within the first 2 years of follow-up to exclude the potential mortality by competing risk, e.g., cancer, etc.

Multiple imputation method was applied to impute the covariates with missing values. All statistical analyses were conducted by using R software (version 4.3.2). The statistical significance was indicated by two-sided *p* value less than 0.05.

## 3 Results

### 3.1 Baseline characteristics of study participants

In this study, a total of 32,698 participants consisting of 2,752 diagnosed with NAFLD and 29,946 without NAFLD were included. The comparison of baseline characteristics between NAFLD and Non-NAFLD (Table 1) showed that a higher percentage of 40–59 age, male, race of Black and White, education level at high school, former or current smokers, ≤ 3.5 and 3.6–4.9 of daily eating frequency, BMI ≥ 30.0, hypertension in NAFLD group compared with Non-NAFLD. The values of ALT, AST, HDL and Serum uric acid of NAFLD were significantly larger than Non-NAFLD.

**Table 1:** Baseline characteristics of study participants.

	Total	NAFLD	Non-NAFLD	*p* value
Participants, No.	32,698	2,752	29,946	
Age, years (%)				<0.001
≤ 39	11,086 (33.9)	827 (30.1)	10,259 (34.3)	
40–59	10,266 (31.4)	1,136 (41.3)	9,130 (30.5)	
≥60	11,346 (34.7)	789 (28.6)	10,557 (35.2)	
Sex (%)				0.02
Male	15,193 (46.5)	1,336 (48.5)	13,857 (46.3)	
Female	17,505 (53.5)	1,416 (51.5)	16,089 (53.7)	
Race and ethnicity				<0.001
Mexican American	5,598 (17.1)	462 (16.8)	5,136 (17.2)	
Non-Hispanic Black	6,575 (20.1)	637 (23.1)	5,938 (19.8)	
Non-Hispanic White	13,953 (42.7)	1,295 (47.1)	12,658 (42.3)	
Other	6,572 (20.1)	358 (13.0)	6,214 (20.7)	
Education level				0.002
<High school	8,476 (25.9)	661 (24.1)	7,815 (26.1)	
High school	7,517 (23.0)	703 (25.5)	6,814 (22.8)	
Some college or above	16,705 (51.1)	1,388 (50.4)	15,317 (51.1)	
Smoking status				<0.001
Never	19,471 (59.5)	1,484 (53.9)	17,987 (60.1)	
Former	7,402 (22.6)	730 (26.5)	6,672 (22.3)	
Current	5,825 (17.9)	538 (19.6)	5,287 (17.6)	
BMI				<0.001
<30.0	20,101 (61.5)	12 (4)	20,089 (67.1)	
≥30.0	12,597 (38.5)	2,740 (99.6)	9,857 (32.9)	
Diabetes				<0.001
Yes	4,274 (13.1)	776 (28.2)	3,498 (11.7)	
No	28,424 (86.9)	1976 (71.8)	26,448 (88.3)	
Hypertension				<0.001
Yes	12,446 (38.1)	1,433 (52.1)	11,013 (36.8)	
No	20,252 (61.9)	1,319 (47.9)	18,933 (63.2)	
Physical activity				0.27
VLPA	987 (5.3)	68 (4.5)	919 (5.3)	
LPA	5,870 (31.3)	457 (30.1)	5,413 (31.4)	
MPA	3,053 (16.3)	259 (17.1)	2,794 (16.2)	
HPA	8,838 (47.1)	735 (48.3)	8,103 (47.1)	
ALT (U/L), median (IQR)	20 (16–27)	26 (19–38)	20 (15–26)	<0.001
AST (U/L), median (IQR)	22 (19–27)	24 (19–31)	22 (19–27)	<0.001
HDL (mmol/L), median (IQR)	1.29 (1.06–1.58)	1.09 (0.91–1.29)	1.32 (1.09–1.6)	<0.001
LDL (mmol/L), median (IQR)	2.90 (2.30–3.52)	2.92 (2.30–3.46)	2.90 (2.30–3.52)	0.90
Serum uric acid (mg/dL)	5.3 (4.2–6.2)	6.1 (5.2–7.2)	5.2 (4.3–6.1)	<0.001

### 3.2 Association between FLI and mortality

**Figure 2:**
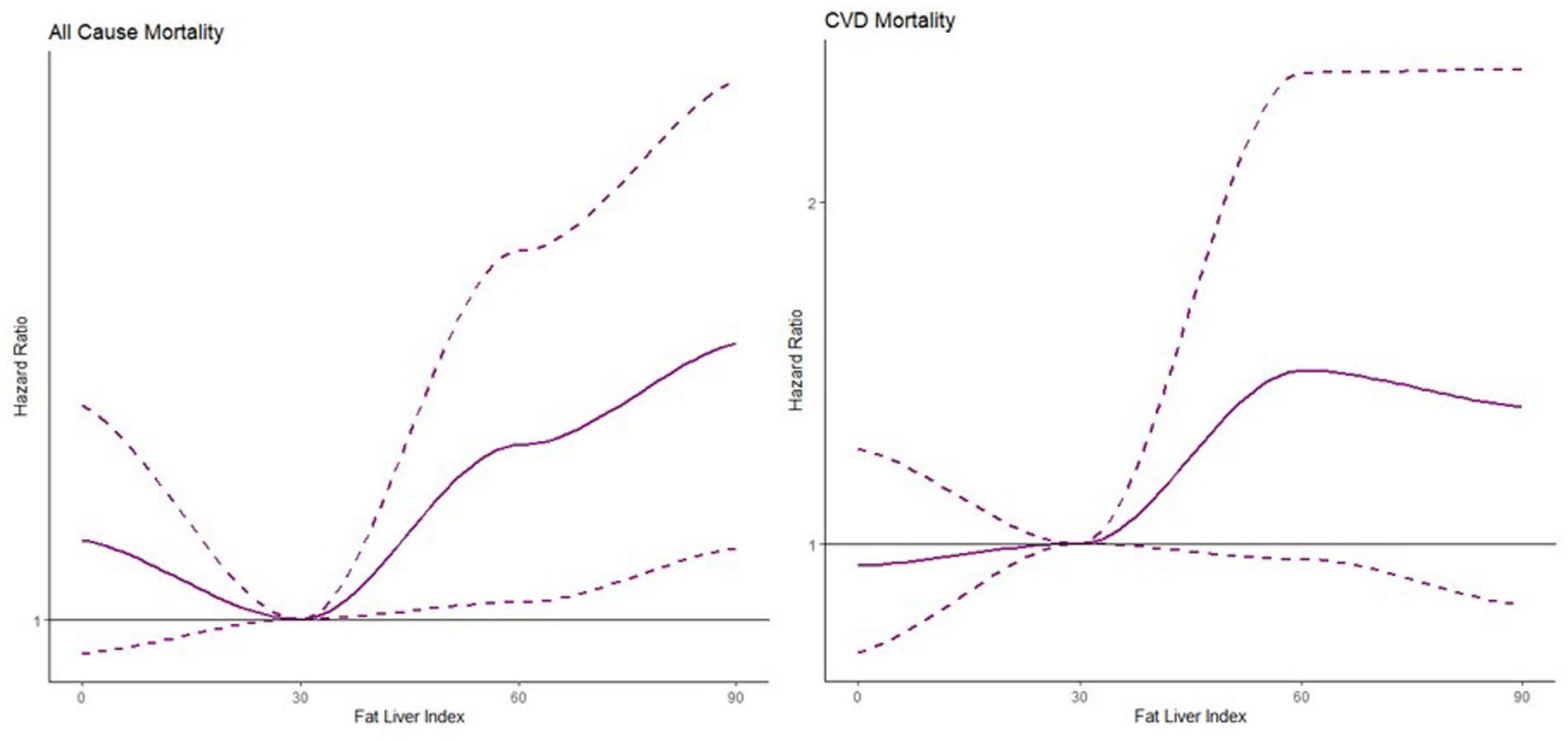
Association between fat liver index and all-cause mortality and CVD mortality.

Based on the three categories by FLI values, the HRs of intermediate state and hepatic steatosis were estimated with no steatosis as the reference level from model 1 to model 3 shown in Table 2. The nonlinear association was found between FLI and all-cause mortality and CVDs mortality in Figure 2. The risk of all-cause mortality was increasing with larger FLI values while the risk of CVDs mortality was the lowest around 30 of FLI and increasing as less or larger FLI.

**Table 2:** Association between FLI and all-cause mortality and CVD mortality.

Categories by FLI	Model 1		Model 2		Model 3	
	HR (95%)	*p*	HR (95%)	*p*	HR (95%)	*p*
All-cause mortality						
No steatosis	Ref.		Ref.		Ref.	
Intermediate state	1.25 (1.03, 1.52)	0.023	1.05 (0.91, 1,22)	0.486	1.14 (0.97, 1.33)	0.110
Hepatic steatosis	1.41 (1.11, 1.79)	0.006	1.31 (1.10, 1.58)	0.003	1.33 (1.10, 1.61)	0.004
CVD mortality						
No steatosis	Ref.		Ref.		Ref.	
Intermediate state	1.60 (1.16, 2.22)	0.004	1.55 (1.11, 2.14)	0.009	1.44 (1.01, 2.04)	0.044
Hepatic steatosis	1.49 (0.93, 2.38)	0.094	1.79 (1.12, 2.88)	0.015	1.65 (1.00, 2.71)	0.049

Model 1: Crude model; Model 2: age and sex included; Model 3: all the significant variables including age, sex, race and ethnicity, education level, smoking status, BMI, diabetes, hypertension, ALT, AST, HDL and serum uric acid value included.

### 3.3 Association between NAFLD and mortality

The comparable mortality rates were noted between the participants of NAFLD and Non-NAFLD. Specifically, the rates of all-cause mortality were 11.3% vs. 11.7% and the rates of CVDs mortality were 3.96% vs. 3.75%, respectively.

The survival analysis was conducted using a weighted Cox proportional hazards regression model. Model 1 was a crude model. In model 2 and 3, except the variable indicating NAFLD or not, for multivariable adjustment, age and sex were included in model 2 and all the significant variables including age, sex, race and ethnicity, education level, smoking status, daily eating frequency, BMI, diabetes, hypertension, serum uric acid group, ALT, AST, HDL, and serum uric acid value were included in model 3. In terms of all-cause mortality, all the HRs of model 1 to model 3 were consistently larger than 1 with statistical significance indicating increased risk in the participants with NAFLD compared to Non-NAFLD. The trend was mirrored in the analysis of CVDs mortality, suggesting NAFLD was associated with an elevated risk of dying from all-cause and cardiovascular causes detailed in Table 3 (39).

**Table 3:** Hazard ratios of NAFLD vs. Non-NAFLD for all-cause mortality and CVD mortality.

Mortality	NAFLD *n*/*N* (%)	Non-NAFLD *n*/*N* (%)	Hazard ratio (95%)	*p* value
All-cause mortality				
Model 1	310/2752 (11.3)	3517/29946 (11.7)	1.31 (1.13, 1.53)	<0.001
Model 2			1.36 (1.16, 1.59)	<0.001
Model 3			1.40 (1.18, 1.66)	<0.001
CVD mortality				
Model 1	109/2752 (3.96)	1122/29946 (3.75)	1.51 (1.18, 1.95)	0.001
Model 2			1.59 (1.24, 2.05)	<0.001
Model 3			1.52 (1.14, 2.01)	0.004

Model 1: Crude model; Model 2: age and sex included; Model 3: all the significant variables including age, sex, race and ethnicity, education level, smoking status, BMI, diabetes, hypertension, ALT, AST, HDL, and serum uric acid value included.

**Figure 3:**
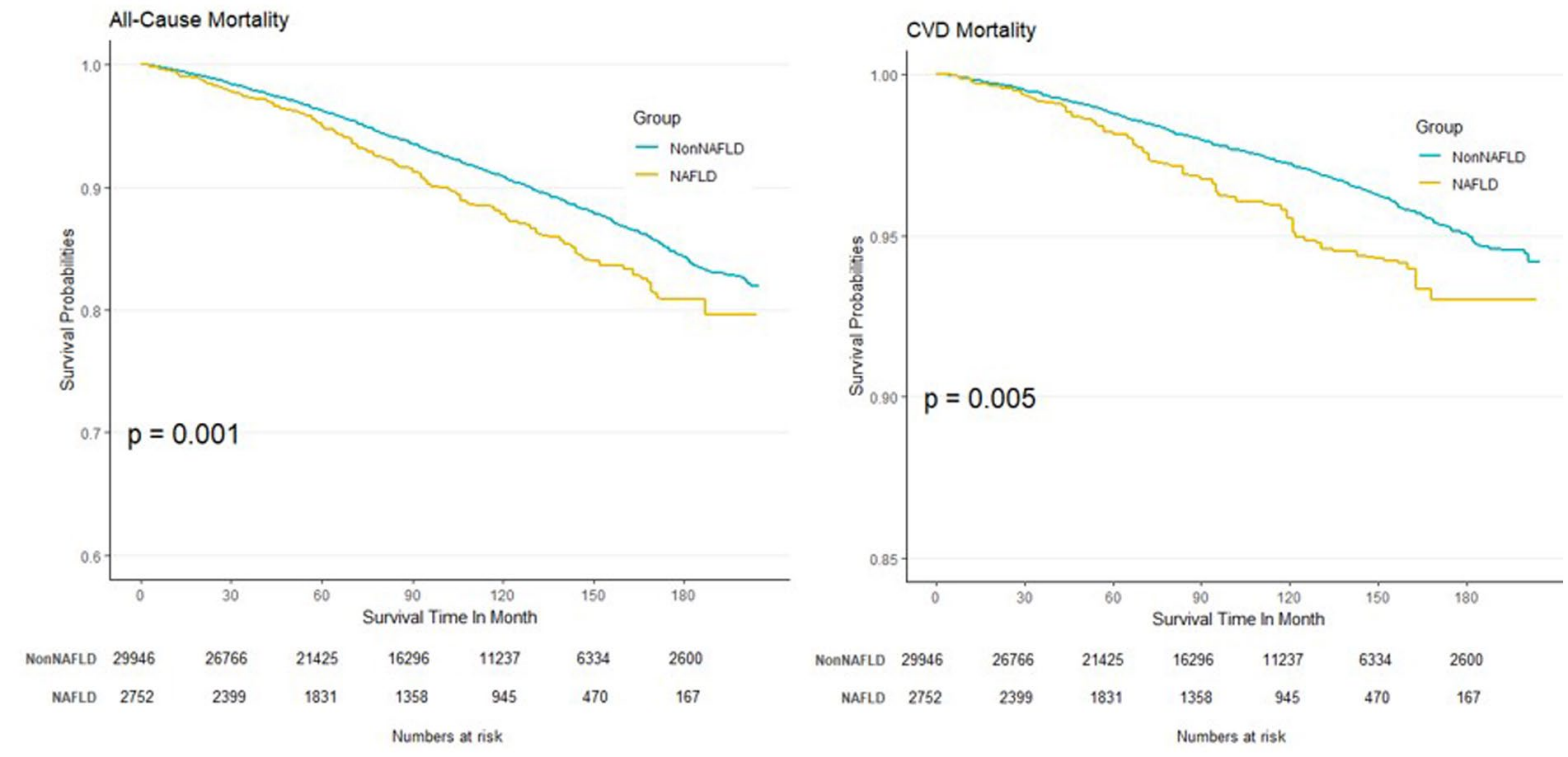
Kaplan–Meier curves between NAFLD and non-NAFLD for all-cause and CVD mortality.

The Kaplan–Meier curves derived from model 1 are depicted in Figure 3. Notably, the curve of CVDs mortality appeared flatter than all-cause mortality due to fewer events cumulated during the follow-up.

### 3.4 Subgroup analyses of the association between NAFLD and mortality

**Figure 4:**
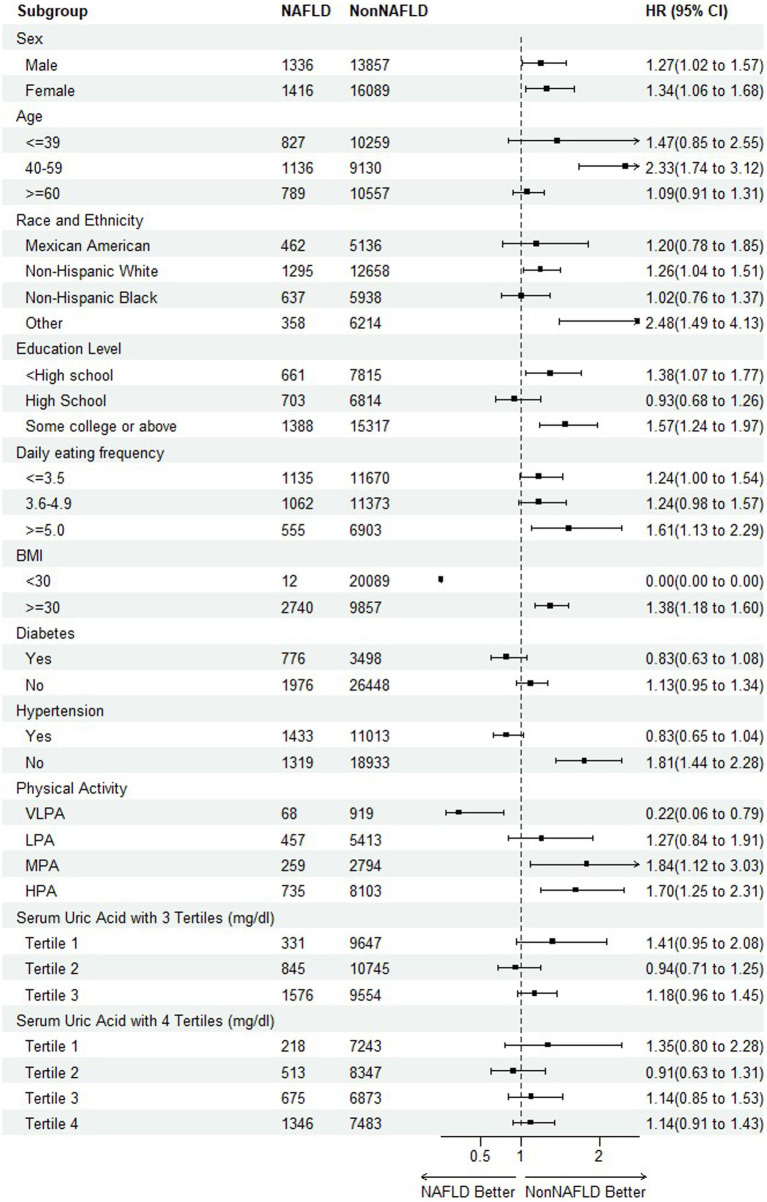
Subgroup analysis of the association between NAFLD and all-cause mortality.

**Figure 5:**
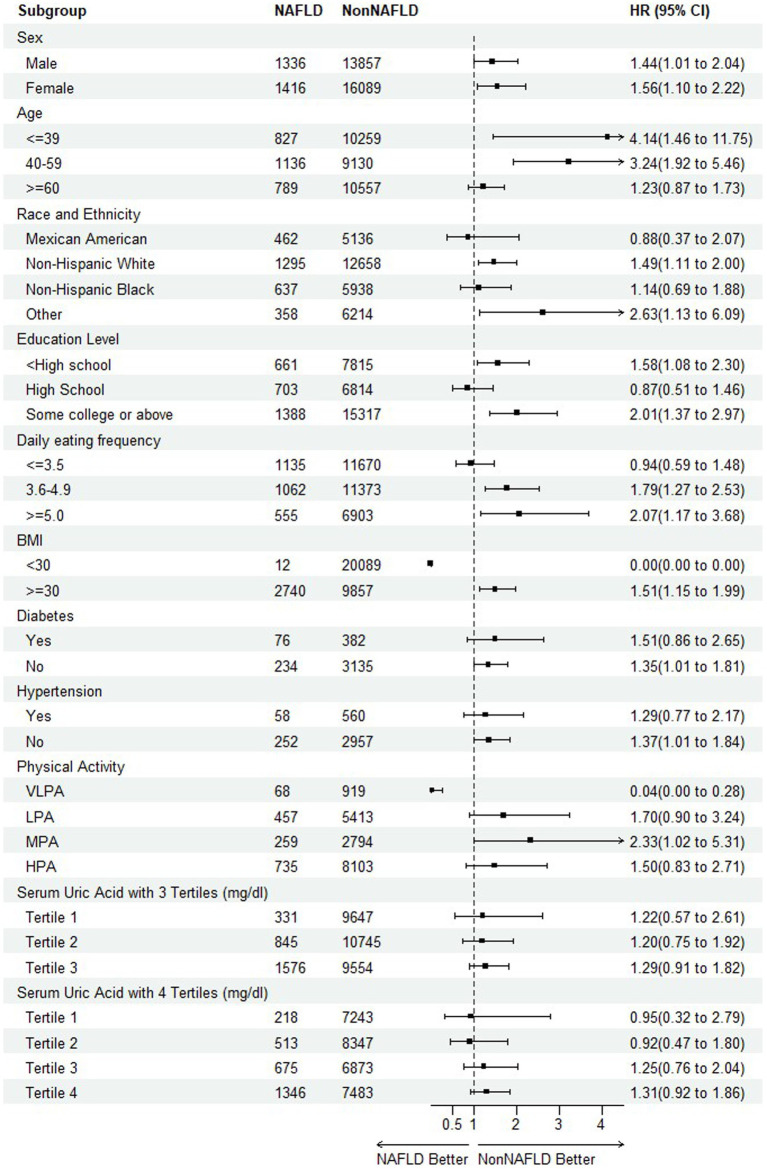
Subgroup analysis of the association between NAFLD and CVD mortality.

To further detect any variability in the impact of NAFLD on mortality based on different baseline characteristics (40, 41), the subgroup analyses were performed on all-cause mortality and CVDs mortality, respectively, by including the risk factors of sex, age, race and ethnicity, education level, daily eating frequency, BMI, hypertension, physical activity, serum uric acid with 3 and 4 tertiles as illustrated in Figure 4, 5.

Regarding the endpoint of all-cause mortality, the statistical significance indicating NAFLD as a risk factor of mortality was observed among the subpopulation of female, aged during 40–59, other race, education level of college or above, 5 or more of daily eating frequence, non-hypertension, physical activity of MPA and HPA. An interesting finding was that as the serum uric acid levels increased, the risk of mortality reached the bottom at tertile 2 before rising again, suggesting a need for further investigation into the relationship. It is also worth noting that in the participants with diabetes or hypertension, HRs were less than 1, although without statistical significance. It might be attributed to its complicated mix of mortality causes and excess mortality irrelated to CVDs occurring in Non-NAFLD group.

For CVDs mortality, with the exception of high school of education level, daily eating frequency (≤ 3.5) and physical activity (VLPA), most HRs supported the trend or conclusion that NAFLD elevated the risk of mortality. Especially among the participants of diabetes and hypertension, the results were consistent among all the sub-levels.

### 3.5 Association between serum uric acid and mortality among the participants with NAFLD

**Figure 6:**
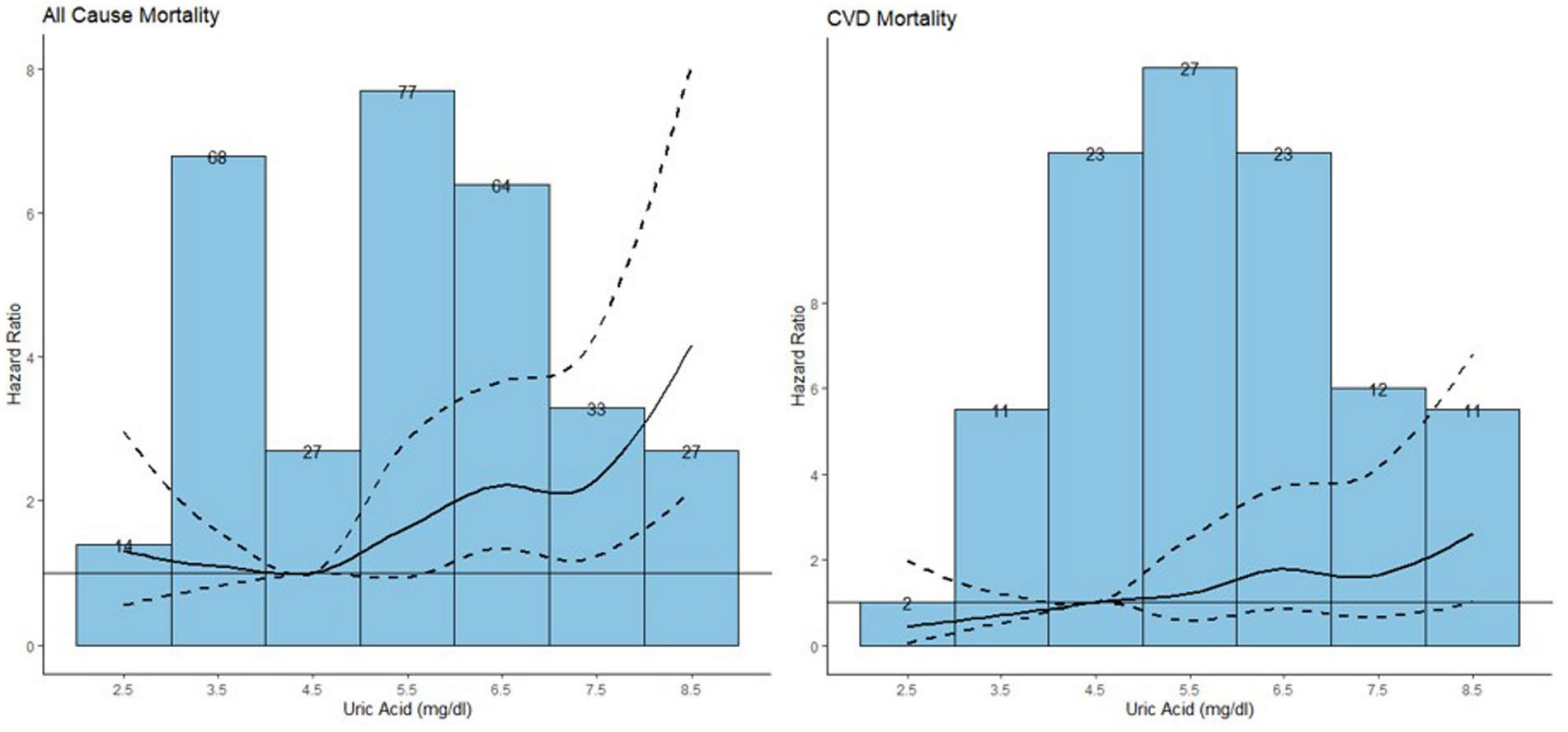
Association between serum uric acid and all-cause mortality and CVD mortality among the population with NAFLD.

To detect the dose–response relationship between SUA and all-cause and CVDs mortality among the participants with NAFLD, we constructed three models by using restricted cubic spline depicted in Figure 6.

The median follow-up duration was 7.25 years with 310 all-cause mortality and 109 CVDs mortality recorded among the participants with NAFLD (42). The HRs and 95% CIs were listed in Table 4. Model 1 is a crude model, model 2 included age and sex as covariates and model 3 included all significant covariates in Table 1. In model 3, an increase of 1 mg/dL in serum uric acid was associated with an 18% increased risk of all-cause mortality [HR (95%): 1.18 (1.07, 1.30)]. For CVDs mortality, while no significant results were observed across various serum uric acid levels relative to the reference level, an increment of 1 mg/dL in serum uric acid was associated with an 22% increased risk of CVDs mortality [HR (95%): 1.22 (1.03, 1.43)].

**Table 4:** Hazard ratios of serum uric acid by tertiles for all-cause mortality and CVD mortality among the population with NAFLD.

Serum uric acid (mg/dL)	Model 1		Model 2		Model 3	
	HR (95%)	*p*	HR (95%)	*p*	HR (95%)	*p*
All-cause mortality						
Continuous tertiles	1.21 (1.14, 1.30)	<0.001	1.17 (1.09, 1.26)	<0.001	1.18 (1.07, 1.30)	<0.001
Tertile 1 (<4.3)	1.21 (0.70, 2.08)	0.489	1.55 (0.89, 2.68)	0.118	1.32 (0.68, 2.57)	0.415
Tertile 2 (4.3–5.3)	Ref.		Ref.		Ref.	
Tertile 3 (5.3–6.2)	1.39 (0.93, 2.08)	0.107	1.30 (0.87, 1.95)	0.199	1.20 (0.76, 1.89)	0.443
Tertile 4 (>6.2)	1.93 (1.35, 2.76)	<0.001	1.81 (1.26,2.59)	0.001	1.51 (0.81,2.81)	0.193
*p* for trend		<0.001		<0.001		<0.001
CVD mortality						
Continuous tertiles	1.23 (1.10, 1.39)	<0.001	1.20 (1.06, 1.35)	0.003	1.22 (1.03, 1.43)	0.019
Tertile 1 (<4.3)	0.43 (0.13, 1.39)	0.159	0.61 (0.19, 1.93)	0.397	0.42 (0.10, 1.79)	0.242
Tertile 2 (4.3–5.3)	0.60 (0.27, 1.32)	0.203	0.66 (0.30, 1.44)	0.293	0.74 (0.34, 1.61)	0.500
Tertile 3 (5.3–6.2)	Ref.		Ref.		Ref.	
Tertile 4 (>6.2)	1.52 (0.85, 2.70)	0.156	1.61 (0.90, 2.88)	0.108	1.97 (0.93, 4.13)	0.075
*p* for trend		0.01		<0.001		<0.001

Model 1: Crude model; Model 2: age and sex included; Model 3: all the significant variables including age, sex, race and ethnicity, education level, smoking status, BMI, diabetes, hypertension, ALT, AST, HDL and serum uric acid value included.

### 3.6 Sensitivity analyses

In the sensitivity analyses, we applied a more rigorous criterion to define NAFLD, i.e., identifying only those with hepatic steatosis (FLI ≥ 60) as NAFLD. Survival analyses were then performed on model 1–3 with their corresponding 95% CIs presented in Table 5. Consistent with the results of Table 3, HRs were all greater than 1 with statistical significance, thereby reinforcing the conclusion that NAFLD was a risk factor for both all-cause mortality and CVDs mortality.

**Table 5:** Hazard ratios of NAFLD vs. Non-NAFLD for all-cause mortality and CVD mortality with FLI > =60 as NALFD.

Mortality	NAFLD *n*/*N* (%)	Non-NAFLD *n*/*N* (%)	Hazard ratio (95%)	*p* value
All-cause mortality				
Model 1	120/1113 (10.8)	3707/31585 (11.7)	1.39 (1.09,1.77)	0.008
Model 2			1.62 (1.26, 2.08)	<0.001
Model 3			1.58 (1.23, 2.04)	<0.001
CVD mortality				
Model 1	40/1113 (3.59)	1191/31585 (3.77)	1.52 (1.01, 2.29)	0.045
Model 2			1.82 (1.21, 2.76)	0.004
Model 3			1.64 (1.06,2.54)	0.026

Model 1: Crude model; Model 2: age and sex included; Model 3: all the significant variables including age, sex, race and ethnicity, education level, smoking status, BMI, diabetes, hypertension, ALT, AST, HDL and serum uric acid value included.

Furthermore, to mitigate the impact of potential mortality due to competitive risks, the participants who died within the first 2 years of follow-up were exclude from the analysis. The resulting HRs and 95% CIs were detailed in Table 6. The results remained consistent with the primary analysis and NAFLD demonstrated a substantial risk factor for all-cause mortality and CVDs mortality.

**Table 6:** Hazard ratios of NAFLD vs. Non-NAFLD for all-cause mortality and CVD mortality after 2-year follow-up.

Mortality	NAFLD	Non-NAFLD	Hazard ratio (95%)	*p* value
	*n*/*N* (%)	*n*/*N* (%)		
All-cause mortality				
Model 1	265/2707 (9.79)	3022/29451 (10.3)	1.30 (1.10, 1.53)	0.002
Model 2			1.33 (1.12, 1.57)	<0.001
Model 3			1.36 (1.13, 1.63)	<0.001
CVD mortality				
Model 1	92/2735 (3.36)	968/29792 (3.25)	1.56 (1.19, 2.05)	0.001
Model 2			1.63 (1.24, 2.15)	<0.001
Model 3			1.51 (1.11, 2.05)	0.008

Model 1: Crude model; Model 2: age and sex included; Model 3: all the significant variables including age, sex, race and ethnicity, education level, smoking status, BMI, diabetes, hypertension, ALT, AST, HDL and serum uric acid value included.

## 4 Discussion

In this large prospective cohort study, NAFLD has been established as a factor associated with increased risk of long-term all-cause and CVDs mortality compared to individuals without NAFLD. After adjusting for a spectrum of relevant covariates including age, sex, race and ethnicity, education level, smoking status, daily eating frequency, BMI, diabetes, hypertension, serum uric acid group, ALT, AST, HDL, and serum uric acid, the substantial association was solidified, providing compelling new evidence for the growing concern surrounding NAFLD.

For the diagnosis of NAFLD, except imaging techniques such as abdominal CT, magnetic resonance imaging (MRI) and liver ultrasonography, a range of non-invasive algorithms were introduced into medical practice (43). The FLI was the one of indexes easily calculated and validated for diagnosis and identifying potential population with NAFLD of future attention (44, 45). Several studies have reported a high degree of concordance between NAFLD diagnosis using FLI and those determined by non-invasive diagnostic markers both biochemical and imaging based. Notably, the agreement was heightened when the intermediate value of FLI, i.e., 30–59 were excluded. Our study revealed the critical finding that hepatic steatosis corresponding to an FLI of 60 or higher was an independent risk factor of all-cause and CVDs mortality. Based on this finding, we conducted a sensitivity survival analysis considering only FLI of 60 or above as indictive of NAFLD and the consistent results were observed. Considering FLI was derived from the variables of triglycerides, gamma-glutamyl transferase, body mass index and waist circumference routinely collected in NHANES, the use of FLI for diagnosis represented an accessible and efficient method.

The association between NAFLD and mortality is complicated and has been a subject of debate (46, 47). For instance, one study involving 619 patients with biopsy-confirmed NAFLD found no difference of mortality between those with and without NAFLD (48). NAFLD is frequently linked to the features of metabolic syndrome and numerous other CVDs risk factors. CVDs has been identified as the leading cause of death among individuals with NAFLD (49, 50). Therefore, in addition to all-cause mortality, to estimate the cause specific association, we also examined the associations between NAFLD and CVDs mortality. Our findings, which controlled for the potential confounders, confirmed the risk association between NAFLD and all-cause and CVDs mortality. The sufficient sample size enabled us to ascertain CVDs mortality attributable to NAFLD with an excess risk rate of 0.2 compared to all-cause mortality.

In the subgroup analysis, homogeneity was observed for the majority of variables, with the exception of a few, e.g., physical activity, hypertension, diabetes for all-cause mortality, and physical activity for CVDs mortality (51, 52). For physical activity, the result at the level of VLPA suggested a paradoxical protective effect of NAFLD, contradicting findings at other levels. This could be attributed to the population with very low physical activity often being older people or those with prolonged bed rest and multiple comorbidities, which could introduce competing risks for NAFLD. Additionally, NAFLD appeared to be protective for all-cause mortality in the population with hypertension and diabetes, potentially due to the introduction of numerous competing risks from various causes of death. This confounding effect was mitigated when focusing on CVDs specific mortality.

Hyperuricemia characterized by the reduction of renal excretion or overproduction of UA, is commonly found in the patients with metabolic disease, which are also the root cause of NAFLD (53). While UA has been associated with NAFLD, its role as a predictive factor remains unconfirmed. The studies by Xinyi et al. indicated that NAFLD patients with elevated UA levels had a relatively higher mortality rate, but UA level in conjunction with NAFLD did not emerge as an independent factor for survival (23). Consequently, we explored the association between UA levels and mortality in the NAFLD participants. Consistent with overall population, the risk reached the lowest level in 4–6 mg/dL for all-cause mortality. For CVDs mortality, the risk increased monotonically with UA levels, diverging from the overall population trend. This discrepancy highlighted the complicated mechanism for CVDs mortality due to the interaction between high FLI and UA levels.

Several limitations should be acknowledged. Firstly, the nature of observation study precluded the establishment of a definitive causal relationship. Secondly, the diagnosis of NAFLD was algorithm-based rather than biopsy-based, which was the golden standard. This could lead to false positive and consequently biased conclusion. Thirdly, given the large sample size and the extended duration of follow up, concerns may arise regarding data quality, including missing values and recalling bias. This suggested our further research would concentrate on conducting exploratory analyses of various NAFLD diagnose approaches, as well as examining subpopulations with some concurrent diseases.

## 5 Conclusion

Non-alcoholic Fatty Liver Disease emerges as a serve metabolic disorder threatening public health, particularly in terms of its association with the increased risk of all-cause and CVDs mortality. Moreover, when NAFLD is comorbid with other diseases, it can lead to an even higher risk of mortality. It emphasizes the need for targeted interventions and preventive strategies.

## Data Availability

Publicly available datasets were analyzed in this study. This data can be found at: National Health and Nutrition Examination Survey (NHANES), https://www.cdc.gov/nchs/nhanes/index.htm.
